# Spoiled breast milk and bad water; local understandings of diarrhea causes and prevention in rural Sierra Leone

**DOI:** 10.1186/1471-2458-13-1172

**Published:** 2013-12-13

**Authors:** Shannon A McMahon, Asha S George, Fatu Yumkella, Theresa Diaz

**Affiliations:** 1 Department of International Health, Johns Hopkins Bloomberg School of Public Health, 615 N. Wolfe Street, Baltimore, MD, USA; 2 Knowledge Management and Implementation Research Unit, Health Section, Programme Division, United Nations Children’s Fund (UNICEF), 3 U N Plaza, New York, NY, USA; 3 Dalan Development Consultants, 12A King Street, off Marcus Jones Estate, Wilberforce, Freetown, Sierra Leone

**Keywords:** Sierra Leone, Diarrhea, Cause, Prevention, Breast milk, Qualitative, Temne, Mende, Water

## Abstract

**Background:**

Globally, diarrhea remains a leading killer of young children. In Sierra Leone, one in seven children die before their fifth birthday and diarrhea is a leading cause. Studies that emphasize the demand-side of health interventions -- how caregivers understand causation and prevention of diarrhea -- have been neglected in research and programming**.**

**Methods:**

We undertook applied qualitative research including 68 in-depth interviews and 36 focus group discussions with mothers, fathers and older female caretakers to examine the causes and prevention of childhood diarrhea in villages near and far from health facilities across four rural districts. Verbal consent was obtained.

**Results:**

Respondents reported multiple, co-existing descriptions of causation including: contaminated water and difficulties accessing clean water; exposure to an unclean environment and poor food hygiene; contaminated breast milk due to sexual intercourse, overheated breast milk or bodily maternal conditions such as menstruation or pregnancy; and dietary imbalances and curses. Respondents rarely discussed the role of open defecation or the importance of handwashing with soap in preventing diarrhea.

**Conclusions:**

Categorizing behaviors as beneficial, harmful, non-existent or benign enables tailored programmatic recommendations. For example, respondents recognized the value of clean water and we correspondingly recommend interventions that reinforce consumption of and access to clean water. Second, respondents report denying “contaminated” breast milk to breastfeeding children. This is a harmful practice that merits attention. Third, the role of open defecation and poor hygiene in causing diarrhea is less understood and warrants introduction or clarification. Finally, the role of exposed feet or curses in causing diarrhea is relatively benign and does not necessitate programmatic attention. Further research supportive of communication and social mobilization strategies building on these findings is required to ensure that improved understanding regarding diarrhea causation translates into improved diarrhea prevention.

## Background

Located on the west coast of Africa, Sierra Leone has an estimated population of 4.9 million [1]. It emerged in 2002 from 11 years of civil war, and today has some of the world’s poorest people and worst health indicators [2]. In 2010, infant mortality was estimated at 114 deaths per 1000 live births and under five mortality was estimated at 174 deaths per 1000 live births, ranking Sierra Leone fifth highest in the world for child mortality [3]. This means that approximately 1 in 11 Sierra Leonean children die before their first birthday, and 1 in 7 die before their fifth birthday [1].

For Africa as a whole, diarrhea is estimated to cause 25% of mortality for children-under-five [4]. In Sierra Leone, it is the leading cause of child mortality [5]. While 82% of rural Sierra Leonean children received ORS or increased fluids as treatment for diarrhea [6], preventive behaviors relevant for diarrhea are low. In rural areas where the most common source of drinking water is surface water (40%), only 4% of households treat their water and the most common type or method of toilet facility is an open latrine (35%) or open defecation (33%) [1].

In April 2010, the government of Sierra Leone introduced free medical care for pregnant women, breastfeeding mothers and children-under-five in order to address, as President Ernest Koroma said, the “deplorable figures of deaths among pregnant women and children” [7]. That same year UNICEF engaged with partners to introduce community case management of diarrhea, malaria and pneumonia in districts with especially poor health indicators. Understanding local illness models is necessary for health professionals to engage with communities in a manner that is respectful, acceptable and appropriate [8]. Applied qualitative research was conducted to provide timely, formative information regarding household understanding of childhood diarrhea, malaria and pneumonia among households located near and far from health facilities across four districts [9]. Research presented here focuses on findings related to diarrhea.

Studies related to the household management of diarrhea peaked in the 1980s and 90s as many governments developed diarrhea control programs [8,10-18]. Recent studies in Mali [18], Tanzania [19] and Kenya [20] explored etiology and management at household and community levels. No published qualitative studies related to diarrhea in Sierra Leone exist [21]. Research findings in this article emphasize household reports regarding diarrhea terminology, causation and prevention.

### Research setting

Research was conducted in four districts of Sierra Leone. Members of the Temne tribe dominate the northern districts Kambia and Tonkolili. Members of the Mende tribe dominate Pujehun District in the south, and Kailahun District in the east. Mendes and Temnes comprise the largest speech communities in Sierra Leone, representing approximately 60% of the total population. Distinctions exist across tribes – particularly a perception that Mendes have had a historical “head start” on development and are favored among “outsiders” [22]. Historians highlight two primary reasons for this situation – the Islamization of the Temne-dominated North, and the Temne’s legendary opposition to colonial authority [22]. Both factors led early Christian missionaries and colonial authorities to favor outreach among Mendes, which led to a proliferation of schools, roads and railways across the Mende South and “gave rise to perceptions of relative deprivation” among Northern groups [22].

Another contextual factor to consider is Sierra Leone’s relatively recent civil war. While the war involved desecration in every district, Southern Mende districts were particularly brutalized. Due to its border location, extensive natural resources (including diamond mines) and status as the “bread basket” of Sierra Leone, Kailahun district was of “central importance” to the Revolutionary United Front (RUF), a rebel army that operated its military bases from Kailahun throughout the war [23]. Kailahun and Pujehun districts became a corridor for logistics and resources between Sierra Leone and Liberia [23]. While bearing the brunt of the civil war, at the close of the war, these districts also received added attention from international relief organizations, which had hosted a broader network of emergency relief operations during the war.

## Methods

### Design and sampling

This qualitative research was embedded within a broader study that purposefully focused on four of the most marginalized districts in Sierra Leone. The qualitative study design was informed by and adhered to principles of applied qualitative research [9,24] that focuses on a specific illness (diarrhea), addresses programmatic concerns related to diarrhea (identifying local causes, terminology), and draws from experiences of actual cases of sick children. Research sought to understand the perspectives of caregivers based in three types of villages: those with a community health center (CHC), those with a health post, and those without any government health facility (located 3-20 miles - or 2-5 hours via foot or transport - from a health facility) with the intention of exploring differences in understandings by distance to facilities. Distinctions have been classified in the data as near village (those with some kind of peripheral government health facility) and far village (remote villages with no government health facilities nearby). Participants were selected with assistance from health centre staff and village aides appointed by the chief; in addition, data collectors canvassed the village and invited those present to participate. Sampling was purposively focused on seeking mothers, fathers and older female caretakers from households that included children under 5 years of age.

### Training

Teams of 4–5 local investigators were trained for five days in sessions that included interview ethics, probing for child illnesses including presumed diarrhea (three or more watery stools a day), piloting interview guides, using tape recorders and writing field notes. Investigators were multi-lingual, college-educated, Sierra Leoneans trained as teachers, nurses, social sciences graduate students, a guidance counselor and a linguist. All investigators had previously engaged in health research. Following training, teams divided by language group and began data collection with a field supervisor.

### Data collection

Two phases of qualitative data collection in April 2010 and July 2010 were undertaken with focus group discussions and in-depth interviews conducted in local languages with mothers, fathers and older caregivers of children under 5 years of age. Following receipt of verbal consent, 36 focus group discussions and 68 in-depth interviews were completed in 12 villages (8 near; 4 far) (See Table 1). Observation guides were also completed in each village describing the availability of health care, water supply and other amenities. Supervisors conducted daily debriefing sessions with interviewers to collectively discuss findings, refine interview guides and identify questions for follow-up interviews. In-country debriefings with national stakeholders following data collection further refined the basis for thematic analysis.

**Table 1:** Respondent groups by district and data collection method

	**Kambia**		**Kailahun**		**Pujehun**		**Tonkolili**		**All districts**	
	***Near***	***Far***	***Near***	***Far***	***Near***	***Far***	***Near***	***Far***	***Near***	***Far***
**In-Depth Interviews (+follow up)**										
*Mothers*	6	4	3(1)	2	16	8	7 (1)	3	32 (2)	17
*Older Female Caregivers*	4 (1)	4	3	1	3	1	2	1	12 (1)	7
									Total = 68 (3)	
**Focus Group Discussions (+ follow up)**										
*Mothers*	2	1	2	1	2	1	2 (1)^a^	1	12 (1)	
*Fathers*	2	1	2	1	2	1	2	1	12	
*Older Female Caregivers*	2	1	2	1	2	1	2	1	12	
									Total = 36 (1)	

^a^All participants returned for a follow-up FGD on the following day.

### Analysis

All interviews were audio-recorded, transcribed into English and assessed for completeness and quality. This study drew upon thematic analysis [25]. A list of hierarchical codes was developed and validated by a co-investigator using debriefing notes collected during data collection and an initial coding of information-rich interviews. Once validated by investigators, the codebook was applied to all transcripts using Atlas/ti [26]. Following this process, codes were grouped into themes related largely to diarrhea terminology, causation and prevention. Across respondent groups, data collection methods and sites, data was compared to arrive at triangulated descriptions of terminology, causation and prevention. Following analysis that emphasized these domains, we sought to prioritize key programmatic interventions into behaviors that are beneficial, harmful, non-existent or benign.

The study received approval from the Government of Sierra Leone Office of Science and Ethics Review Committee, Ministry of Health.

## Results

### Local terminology and classification

Respondents identified a range of terms for diarrhea outlined in Krio, Mende and Temne in Table 2. English is the official language of Sierra Leone and Krio is the *lingua franca*, but in Pujehun and Kailahun, Mende is generally spoken; in Kambia and Tonkolili, Temne is generally spoken. While we are not seeking “equivalence” [27], we document in Table 2 the terms *most commonly used* by mothers to describe general-to-severe diarrhea in their children.

**Table 2:** Local terminology and classification for diarrhea

**Term**	**Language**	**Meaning**	**Context**
*Run belleh*	Krio	Running stomach	Most common term for diarrhea, nationwide
*Wata wata kaka*	Krio	Water water feces	Common diarrhea
*Kohunlea*	Mende	Watery stool	Most common term for diarrhea in Mende
*Kohunlea gbandi gbandi*	Mende	Watery stool hot hot	Severe diarrhea (in Kailahun district)
*Gboio-laa koehun*	Mende	Incessant stool	Severe diarrhea (in Pujehun district)
*Dengbei*	Mende	Slimy stool sometimes with blood	Severe diarrhea (in Pujehun and Kailahun districts)
*Nja-norhuin*	Mende	Dirty water	Common term for diarrhea in Mende
*Kabom*	Temne	Defecation	Common term for diarrhea in Temne
*Kagboshea*	Temne	Frequent stool	Common term for diarrhea in Temne
*An runt*	Temne	Diarrhea sometimes with blood	Severe diarrhea (Kambia districts)
*Cholera*	English		Severe diarrhea, sometimes with vomiting (all districts)

### Diarrhea causes and prevention

Most respondents identified diarrhea as a major illness affecting children. Terms used to describe diarrhea in children are presented in Table 2. Respondents also elaborated on causes of diarrhea and, upon probing, prevention mechanisms. Sometimes respondents mentioned treatment measures such as ORS and antibiotics as their main prevention strategy. In some remote villages in Tonkolili and Pujehun districts, mothers reported that they had no understanding of diarrhea etiology.

During analysis, causes related to diarrhea were grouped into categories that emerged from the data: contamination, spoiled breastmilk, actions of an external agent and imbalance (see Tables 3 and 4) [8]. Tables are organized by distance to facility, respondent type and data collection method. Tables also highlight sub-bullets outlining facets of each broad category. “Contamination” encompasses contaminated food or water, contamination from insects, exposure to a “dirty” environment, or poor personal hygiene (of caretaker or child). “Spoiled Breastmilk” refers to contaminated milk of a lactating mother, a cause of diarrhea that was mentioned across respondent groups, in interviews and discussions, regardless of distance to facility or district of residence. “Actions of an External Agent” were mentioned rarely but refer to external curses on a mother or her ailing child, the spirits of angry ancestors or the will of God. An “Imbalance” refers most commonly to a dietary imbalance such as consuming too much or too little of a certain food, but also includes weaning or a sudden change in diet.

**Table 3:** Causes of diarrhea as reported* by Mothers (M), Fathers (F) and Older Female Caretakers (C) in villages far (≥ 3 miles) from health facilities across 4 districts in Sierra Leone

		**Kambia**	**Tonkolili**	**Pujehun**	**Kailahun**
***Diarrhea Causes***					
**1.0 Contamination**					
1.1 Insects transferring germs through bites or landing on food	Flies (transfer material from feces to food/nipple)	**F, C**	**C,***M*	**M,***M*	**C**
	Worms (in child’s stomach)				**F**
1.2 Exposure to contaminated physical environment	Playing in dirt, child playing with own feces, sharing a toilet with an individual who has diarrhea			**F**	
	Exposure to contaminated food and utensils (uncooked meat or fish, uncovered food, eating food off the ground)	**F, C**			**M, C**
	Exposure to contaminated drinking water	**M, F, C**	**C, F**	**F, C**	**M, F, C**
			*M, M, C*	*M, M*	
	Exposure to contaminated nipple (sweaty, dirty)				**F**
1.3 Poor hygiene	Handfeeding	**C**		**M**	**M, C**
	Unwashed hands of either child or mother (without mention of handfeeding)				
	Wearing dirty clothes (child)				**C**
**2.0 Spoiled Breastmilk**					
2.1 Exposure to contaminated breast milk	Contamination due to sexual relations	**M, C**	**M, F, C**		**M, F, C**
		*M*			
	Contamination due to overheated milk in breasts/contaminated breast	**M, C,**	**M. F**	**C**	**F**
		*M*			
	Contamination due to a mother breastfeeding while pregnant/menses		**M**		
	Contamination due to eating foods considered inappropriate for breast feeding mothers	**M**	**F,***M*		
**3.0 Action of External Agents**	Malevolent Sprits, witches, angry ancestors, the will of God	**M**	**F**	**C**	**C**
		*M, M*			
**4.0 Imbalance**					
4.1 Dietary	Eating too much of a certain food or a bad mix of food	**M,***M,*		**M**	*M*
	A sudden change in child’s diet (i.e. children eating new foods when they begin being cared for by grandmothers)				**F, C**
					*M*
	Weaning	**C**			**C**

*Respondent indicators from FGDs are in bold text, respondent indicators from IDIs are *italicized*. Data collected in 2010.

**Table 4:** Causes of diarrhea – as reported* by Mothers (M), Fathers (F) and Older Female Caretakers (C) in villages near (< 3 miles) health facilities across 4 districts in Sierra Leone

		**Kambia**	**Tonkolili**	**Pujehun**	**Kailahun**
***Diarrhea Causes***					
**1.0 Contamination**					
1.1 Insects transferring germs through bites or landing on food	Flies (transfer material from feces to food/nipple)	**M, F, C**	*C*	**F**	**M, F, C**
				*M, M*	
	Worms (in child’s stomach)	**M, C**	**C**		**C**
1.2 Exposure to contaminated physical environment	Playing in dirt, child playing with own feces, sharing a toilet with an individual who has diarrhea	**M**	**F,***C*	**M, C**	**M**
				*M*	
	Feces near homes		**M**	**F**	**F**
	Exposure to contaminated food and utensils (uncooked meat or fish, uncovered food, eating food off the ground)	**M, F, C,**	**M, F, C,**	**M, F, C**	**M, F,**
		*M, M*	*C*		*C, M*
	Exposure to contaminated drinking water	**M, F, C**	**M, F, C**	**F, C**	**M, F**
		*M, M*	*M, M, C*	*M*	
	Exposure to contaminated nipple (sweaty, dirty)		**M**		**M, F, C**
					*M*
1.3 Poor hygiene	Handfeeding	**C**		*M, M*	**M, F,***M*
	Unwashed hands of either child or mother (without mention of handfeeding)	**F, C**	**M, F**		**M**
	Wearing dirty clothes (child)	*M*			
**2.0 Spoiled Breastmilk**					
2.1 Exposure to contaminated breast milk	Contamination due to sexual relations	**F**	**F, C**	**M**	**M, F, C**
			*C*		
	Contamination due to overheated milk in breasts	**M, F, C**	**M, F, C,***M*	**F**	**M, F, C**
		*M, M, C*			
	Contamination due to a mother breastfeeding while pregnant/menses				**M**
**3.0 Action of External Agents**	Malevolent Sprits, witches, angry ancestors, the will of God	**C**	**M, F, C**	**C**	*C*
				*M*	
**4.0 Imbalance**					
4.1 Dietary	Eating too much of a certain food or a bad mix of food	**M, F, C**	**M**	**M,**	**M, F**
		*M, M*		*M*	
	A sudden change in child’s diet (i.e. children eating new foods when they begin being cared for by grandmothers)	**M**			
	Weaning	*M, C*		**M,***M*	**M**

*Respondent indicators from FGDs are in bold text, respondent indicators from IDIs are *italicized*. Data collected in 2010.

Contamination was mentioned as the primary cause of diarrhea in nearly all focus groups and interviews. In villages far from a facility, the two primary causes of contamination include contaminated water and contaminated breastmilk due to sexual relations. In villages near a facility the primary causes of diarrhea include: contaminated food or utensils; contaminated drinking water; breastmilk contaminated primarily via overheating but also via sexual relations; and eating too much of a certain food or a bad mix of food.

### Contamination

#### Contaminated water and poor hygiene

Contaminated water was discussed as a cause of diarrhea across all respondents groups, villages and districts (Tables 3 and 4). Contaminated water was described as water from a stream or river, water that is not covered, and water that has a “taste”, with the understanding that tasteless water is clean. Many respondents were able to distinguish between different kinds of water, prioritizing tap or pump water as being cleaner than water collected from streams or uncovered wells.

Here we have tap water, well water and river water. If you get from the river and the water did not fit you. You will *run belleh* … because they do a lot of things there. They do toilet and launder there. -Interview Mother, Tonkolili District, Near Village

Despite knowing that pump and tap water is better, respondents said that at times they did not have a choice about their source of water due to problems with pumps and taps, contamination during rainy seasons or scarcity during dry seasons.

If there is no pure drinking water, like it happened yesterday when the tap got spoiled, there is no other means of getting water except the water we get from the farm … that will lead to *khohunlei (diarrhea).* -Focus Group, Mothers, Kailahun District, Far Village

With the exception of remote villages in Kambia and Kailahun, respondents were more likely to discuss poor hygiene such as serving food with contaminated utensils, eating food that was unwashed and hand feeding in villages with health facilities. While hand feeding was mentioned by respondents as a cause of diarrhea and hand washing was discussed at least once in most districts as a preventive measure, hand washing was rarely linked to hand feeding and never mentioned as a precaution caretakers should take before preparing food.

The other has to do with leaving your child’s food open. Hence houseflies would defecate on the food and it would affect the child if he/she eats it. Secondly you should not use your hand to feed your child. You should use a spoon to feed your child. -Focus Group, Grandmothers, Kambia District, Far Village

The role of flies in transmitting germs from feces to food or utensils was mentioned by at least a few respondents across all districts (Tables 3 and 4). Mothers in Pujehun and Kailahun reported the highest degree of detail on the role of flies and often attributed their knowledge to “the nurses” who “come around and talk to us” during vaccination campaigns. While feces near homes was listed as a cause of diarrhea in villages with health facilities, it was not mentioned by any respondents in distant villages (Tables 3 and 4). Similarly, while children playing in dirt, coming into contact with feces or sharing a toilet with someone who had diarrhea was mentioned as a cause of diarrhea in villages with health facilities, this was only mentioned once in a remote village by fathers in a focus group discussion in Pujehun. Worms were also identified as a cause of diarrhea in villages with facilities, but not by those in distant villages.

After discussing causes of diarrhea, respondents were probed with regards to ways of preventing diarrhea. Several conversations mentioned the need to consume clean water, to prevent children from playing with their own feces, and to prevent flies from transferring “germs” or “fly feces” onto the child or child’s food. At least one group of respondents across districts discussed keeping children’s feet covered to prevent illnesses including diarrhea, others mentioned that contamination could occur by smelling feces.

Respondent 1: It is advisable to be cleaning our compound, and that when the child goes to toilet you must throw it away or make sure that it is covered, when this is done it can prevent the child from having *run belleh (diarrhea)*

Respondent 2: If you are living in a place that is dirty and you just leave your child to walk around barefooted and dirty, but if in the morning you washed you child and dressed him nicely with foot wear on it will also prevent *run belleh*.

-Focus Group, Mothers, Kailahun District, Near Village… the dirt. At times even its odor can disturb. When children go around to play with the dirt and the heat in that dirt goes up their head they are bound to become sick.

-Focus Group, Mothers, Tonkolili District, Far Village

In Kambia and less so in Kailahun and Pujehun, caregivers reported a sense of futility in avoiding childhood diarrhea - because it is an unavoidable fact of childhood, because children’s behavior cannot be guarded at all times or because parents have no choice but to subject their children to contaminated environments or water.

Another cause of cholera is when the baby is not cared for. Sometimes when the baby is crawling and its hands may get dirty and we all know babies put almost anything in their mouths. Or even if they end up eating food with these dirty hands, they will get cholera.

- Focus Group, Fathers, Kambia District, Near Village

### Spoiled breastmilk

Along with contaminated water, a contamination that was discussed in detail across respondent groups in all districts (but less so in Pujehun) was spoiled breastmilk. In particular, sexual intercourse causing breast milk contamination was discussed extensively across respondent groups regardless of district of residence or distance to a health center and in both FGDs and IDIs (see Tables 3 and 4). In Mende, the word *pohunlay* literally means “jumping over the foot of the baby” and refers to having sexual intercourse while a child is still being breastfed. Weaning was understood by respondents as being from 1½ to 2 years of age or until the child can walk. Some older female caregivers talked about a generational shift, wherein older generations were more capable of abstinence after childbirth than younger generations who resume intercourse as soon as 40 days after birth. Mothers who are breastfeeding come under pressure in the event that their infant presents with diarrhea, as the diarrhea is a public sign of her immorality (according to fathers and older female caretakers) or her inability to stop the advances of her husband (according to mothers themselves).

Sometimes, when the woman is lactating, the man will not bear up but he will ask and ask the mother to go to bed…this leads to the *or bom* (the baby toileting).- Interview, Mother, Kambia District, Far Village

Furthermore, fathers raised diarrhea as a sign of infidelity in contrast to mothers or older caregivers.

Even if she does it with her husband - when she does it with another man, and the husband does not take notice of that, the child makes other people take notice, through the diarrhea. -Focus Group, Fathers, Kailahun District, Far Village

Mothers alone mentioned breast milk getting spoiled due to the heat caused by sex, or heat from the sun or heat generated internally during long days of farm work.

Respondent 1: If you leave your child for a whole day and you just give the breast to your child, the child will toilet for this. Because the impure and pure breast will mix together and this can cause the child to toilet.

Interviewer: So because you left the child the whole day and then when you return you give the child your breast?

R1: Without washing it.

I: So it will be a bad one? The breast will spoil?

R2: You have been out for a whole day with sweat, so that will cause the child to toilet.

R1: If you spend the whole day you did not give the breast to your child and you later come and give the warm breast to the child. The child will toilet.

-Focus Group, Mothers, Tonkolili District, Far Village

Breastmilk was also reportedly tainted if a mother herself was pregnant, menstruating or sick. A mother could also contaminate her breast milk by drinking contaminated water or forbidden foods.

Interviewer: Which sickness does a breast have?

Respondent: It has those *tumbu* (germs).

I: Can you tell me about those *tumbos?*

R: Yes the *tumbos* are inside the water, when the *komra* drinks the water.

-Interview Mother, Tonkolili District, Far Village

Respondents discussed various ways to prevent diarrhea due to spoiled breast milk consumption, which included expelling hot breast milk prior to feeding, rubbing contaminated breasts with herbs and administering medicinal herbs or special foods to a breastfeeding mother.

One of the ways is that after a whole day of work by the mother, when she come back home, she should spill the first breast milk, by so doing the hot milk will be thrown and a cold one will come and the child will suck it. -Focus Group, Fathers, Kailahun District, Far Village

Sometimes some people will pick paw-paw leaves and some medicinal herbs … to cure the frequent stooling. This medicine is not given to the children but to the suckling mother. Sometimes the breast milk becomes sour and the child refuses to suckle the breast until the mother takes this cure, the child will not suckle.

-Interview, Mother, Kambia District, Far Village

Although two respondents mentioned breastfeeding and particularly exclusive breastfeeding during the first six months of life as an important action for children with diarrhea, they were in the extreme minority. More commonly the response to contaminated breastmilk, particularly if affected by sexual intercourse, was to stop breastfeeding the child.

Respondent: (long pause) Well … I have stopped breast feeding her.

Interviewer: You had stopped breast feeding her ehhh.

R: Because others are saying it is my breast.

-Interview, Mother, Kambia District, Near Village

### External agents

The role of curses on a mother causing diarrhea in her child emerged regardless of village location. It was discussed in detail in Temne districts, Kambia and Tonkolili. By contrast, in Mende districts of Pujehun and Kailahun, mainly older female caretakers mentioned the role of supernatural forces causing diarrhea. In the case of curses, pregnant women who become cursed are banned from eating certain foods, or eating food cooked in the same fire as banned foods. If a ban is violated, a woman’s unborn child or breastfeeding infant will be destined to experience diarrhea. Banned foods in Tonkolili included cassava leaves, tortoise, ducks, chickens and eggs. Eggs were also mentioned as a banned food in Kambia. A woman can become cursed due to her bad attitude, witnessing a men’s-only ceremony, being unfaithful or being generally unfortunate. In no conversation were curses on men mentioned.

Respondent 1: it is our belief that, during pregnancy the stomach of the pregnant woman will cause noise known as *anfutha (curse)*. When this happens the woman is taken to a special traditional ceremony (*anya-gbeh)…* [where] the women is advised not to eat eggs and chicken.

Interviewer: How does this curse of *kabom (diarrhea)* come then?

R1: When she mistakes to eat either of the two. It is a belief that the unborn child will have *kabom*.

-Focus Group, Older Female Caretakers, Tonkolili District, Near Village

### Dietary imbalance

Dietary imbalances were mentioned in all districts, but less in Tonkolili and Pujehun. Eating too much of a certain food (mangoes or oranges) was discussed in depth in Kambia district, especially in villages with a health facility. Changing a child’s diet due to weaning from breast milk and therefore introducing milk, tea, eggs or fish was identified as causing diarrhea. In a few villages, changes in diet related to being taken care of by older caregivers was also mentioned as a cause of diarrhea.

## Discussion

Diarrhea causes and prevention as described by mothers, fathers and older female caretakers of children under five years of age include contamination, spoiled breastmilk, actions of external agents and dietary imbalance. Contamination of water, food or breast milk was discussed in the greatest depth. Distinctions across respondents were stronger comparing across districts rather than by distance to a health facility.

Speaking broadly, respondents across Kailahun demonstrated the strongest understanding of the link between contaminated water and poor hygiene leading to diarrhea. At least some respondents in all districts, whether near or far from a health facility, could detail the ways in which water from an unprotected source caused diarrhea. However, pragmatic limitations impeded this process; for example, tap water was physically inaccessible or children could not be prevented from drinking river water. Contamination of breast milk was discussed in greatest detail in Kambia, followed by Tonkolili and Kailahun. The role of sprits or external agents causing diarrhea emerged most strongly in Temne districts of Kambia and Tonkolili.

Across districts, the concept of prevention was sometimes difficult to convey and explore with respondents regardless of their distance to a health facility. While we have presented how households discussed causes and responses with regards to preventing diarrhea in children by separate categories, often these concepts were discussed simultaneously. It was not uncommon for biomedical prevention concepts linked to hygiene, clean water and sanitation to co-exist with prevention measures linked to abstaining from sex while breastfeeding and protecting lactating women from evil spirits.

Our findings correspond with studies across sub-Saharan Africa countries related to diarrhea causation including the role of a dirty environment and polluted water [10,13,28], bad food or an imbalanced mix of food [16-18], various insects including flies, mosquitoes or worms [12,29,30], supernatural forces such as witches or religious spirits [10,30,31] and spoiled breast milk. Spoiled breast milk emerged as especially relevant in this context, similar to studies exploring how a mother’s sexual behaviors led to childhood diarrhea in Ghana [32], Zimbabwe [10,32], Nigeria [33,34], Burkina Faso [14], Mali [14,18], Tanzania [19] and Kenya [20]. Teething or the emergence of tooth buds, while reported in several studies across sub-Saharan Africa [10,11,13,17,28,35,36], was scarcely identified as a cause of diarrhea in Sierra Leone.

Following frameworks of behavior change [37] and models for community based research [38], opportunities for programmatic interventions can be classified into four strata (beneficial, harmful, incomplete or benign) that build on existing understandings, rather than primarily assume that communities lack awareness. Some understandings can be classified as desirable and beneficial and could be reinforced; others can be classified as undesirable and harmful and should be targeted for change; some do not exist or are incomplete and merit introduction or clarification; finally some are benign and require little attention.

In the first strata, we place respondents perceptions regarding the lack of availability of clean water and ubiquity of contaminated water leading to diarrhea. Since many respondents draw a link between these factors, there are favorable conditions to pursue, namely structural interventions to improve infrastructure coupled with behavioral interventions that aim to stimulate a collective decision at the level of the village to support access to clean water, end open defecation and, as part of the process, improve awareness and knowledge regarding preventive practices such as treating impure water and improving hygiene. Existing experience on ending open defecation through the Community Approaches to Total Sanitation within Sierra Leone as well as in other countries can be readily applied in the study villages to foster sustained changes in social norms.

In the second strata, we note an understanding that “spoiled breast milk” causes diarrhea. This contravenes a biomedical understanding that continued breastfeeding is crucial to the development of young children, in particular those suffering from illnesses such as diarrhea. It merits active outreach to address current understandings with mothers and fathers through child health and nutrition activities, but also through family planning and maternal health services.

In the third strata, we note that - with the exception of Kailahun, few respondents mentioned a link or understood the mechanisms between open defecation, flies, hand feeding and washing frequently and especially at crucial moments (after defecation, after cleaning a child’s bottom or before preparing/serving food) [39]. This presents a further opportunity to support interventions, including those involving activities to foster positive social norms regarding community sanitation and personal hygiene. As a final point, findings such as the role of an imbalanced diet, diarrhea entering a child’s body through their feet or curses causing illness are relatively benign and are therefore a low priority for public health interventions.

### Limitations

While in-depth interviews provide illness narratives, they still reflect retrospective reported practice, which may be different from the pragmatic realities that become apparent when observing child health care episodes or tracking them prospectively. Exploring multiple child illness concepts in focus group discussions in some instances led to informant fatigue, as a result of which not all topics were probed evenly. Furthermore, language barriers among older female caretakers in one village in Tonkolili forced an early end to data collection in that village. Finally, while data collection teams were comprised of graduate-level, multilingual data collectors, variations in level of ease (and comfort) speaking in dialects, collecting data and interviewing respondents on health-related material in hardship settings existed. We sought to mitigate this limitation via trainings and daily debriefings led by a public health researcher who accompanied teams throughout data collection.

## Conclusion

Respondents in Sierra Leone are not 'empty vessels’, wholly unaware of what causes diarrhea [9,40]. They have multiple, co-existing descriptions of causation, which informs their understandings regarding prevention and illness response. A nuanced approach that categorizes behaviors as beneficial, harmful, non-existent or benign is warranted. Community engagement processes where members collectively assess diarrhea causes and develop responses that support pro-hygiene messaging and social rewards (or sanctions) for those who do (or do not) adopt beneficial behaviors can foster individual and collective responsibility for diarrhea prevention. Understanding who are trusted sources of information, how knowledge is communicated and reinforced and what factors may cue behavior change can further aid prevention strategies. Campaigns that assume a universal lack of awareness or emphasize blanket health education messaging are not recommended.

## Competing interests

The authors declare that they have no competing interests.

## Authors’ contributions

SM executed this study, conducted analysis and wrote this manuscript. AG designed the study, made critical edits to tools, participated in analysis and edited this manuscript. FY oversaw data collection and edited this script. TD designed the study and edited this manuscript. All authors read and approved the final manuscript.

## Pre-publication history

The pre-publication history for this paper can be accessed here:

http://www.biomedcentral.com/1471-2458/13/1172/prepub
